# *QuickStats:* Percentage* of Children and Adolescents Aged ≤17 Years Who Used Telemedicine During the Past 12 Months,^†^ by Age Group and Year — United States, 2021–2023

**DOI:** 10.15585/mmwr.mm7335a4

**Published:** 2024-09-05

**Figure:**
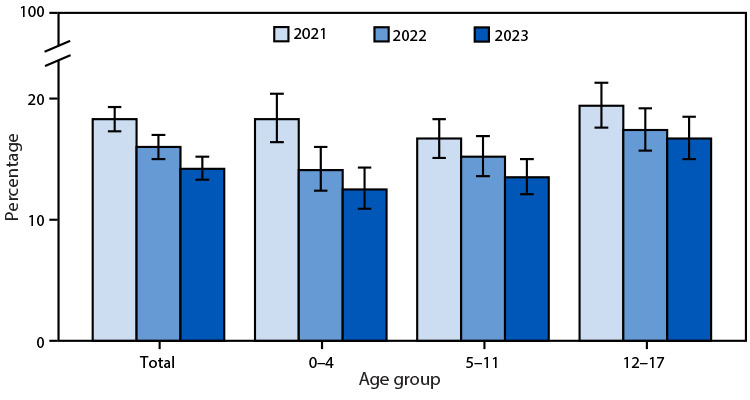
The percentage of children and adolescents aged 0–17 years using telemedicine during the past 12 months declined from 18.3% in 2021 to 14.2% in 2023. Telemedicine use declined across all three age groups during this period. In both 2022 and 2023, telemedicine use increased with age.

**Supplementary Table:** https://stacks.cdc.gov/view/cdc/159160

**Source:** National Center for Health Statistics, National Health Interview Survey, 2021, 2022, and 2023. https://www.cdc.gov/nchs/nhis.htm

